# Genetic Diversity of Rose Rosette Virus Isolates in Oklahoma Reveals a Distinct Evolutionary Lineage

**DOI:** 10.3390/v18101069

**Published:** 2026-09-26

**Authors:** Caleb Paslay, Akhtar Ali

**Affiliations:** Department of Biological Science, Oxley College of Health & Natural Sciences, The University of Tulsa, Tulsa, OK 74104, USA; cap1050@utulsa.edu

**Keywords:** rose rosette virus, phylogenetics, population dynamics, genetic diversity, molecular evolution

## Abstract

Rose rosette virus (RRV), a member of the genus *Emaravirus*, is responsible for rose rosette disease (RRD), which has significant impacts on the rose industry and ornamental landscapes across the United States (US). To better understand the genetic diversity and population structure of RRV, infected rose leaf samples were collected from garden centers, nurseries, retail stores, and wild rose populations. Total RNA was extracted and analyzed using Sanger sequencing and Illumina-based high-throughput sequencing (HTS), resulting in nearly complete genome sequences of 17 RRV isolates. Phylogenetic and population genetic analyses incorporating >100 publicly available RRV isolates revealed a genetically differentiated lineage comprising eight isolates, seven of which were characterized in this study. This lineage, designated the OK Clade, was consistently supported across the concatenated coding-sequence dataset and several individual genomic segments of RRV. Evolutionary modeling estimated that the divergence between the OK Clade and all other RRV isolates likely occurred more than a century ago. Population genetic analyses further supported substantial differentiation between the OK Clade and other RRV isolates. RRV exhibited greater nucleotide diversity than previously reported, indicating that the genetic diversity of the virus may be broader than previously recognized. Potential recombination breakpoints were also identified in RNA1 and RNA5, although additional analyses are required to validate these events. Collectively, these findings identify a geographically associated and genetically distinct RRV lineage in Oklahoma and provide new insights into the population structure and evolutionary diversity of RRV.

## 1. Introduction

*Emaravirus rosae* (Genus: *Emaravirus*, Family: *Fimoviridae*), commonly referred to as rose rosette virus (RRV), is a significant viral pathogen affecting the rose industry and ornamental landscapes. RRV is the etiological agent of rose rosette disease (RRD), a condition that remained of unknown origin for several decades. Although RRD has been documented for more than 80 years, RRV has only been identified as its causal agent within the past 15 years [1].

The presence of RRV is typically characterized by the development of distinct symptoms, which may appear within 1–6 months following initial infection or after the conclusion of winter dormancy [2,3]. These symptoms include rosette (witches’ broom), persistent reddening, excessive thorn production, deformation of leaves and flowers, and mosaic patterns on foliage.

RRV inflicts significant damage and diminishes the aesthetic value of roses. For instance, the rose industry in the United States (US) has experienced revenue losses of approximately 25% attributable to this disease [3]. In Tulsa, Oklahoma, the Tulsa Rose Gardens were severely affected by RRD, resulting in the removal of hundreds to thousands of rose plants [4]. Current evidence indicates that RRV is largely confined to the North American continent, with widespread reports across the U.S. Notably, more than 75% of the U.S. states have documented at least one occurrence of RRV (https://roserosette.org/distribution-map/, accessed on 1 May 2026) based on collaborative reporting efforts from researchers and the public. RRV has been reported predominantly in the United States, with an additional report from India [5].

The RRV genome comprises seven segments of negative-sense RNA. Initial studies of the virus identified only four genomic segments, designated RNA1–RNA4, which constitute the conserved core RNAs shared among members of the genus *Emaravirus* [1,6]. The core segments encode the RNA-dependent RNA polymerase (RdRP), glycoprotein precursor (GPP), nucleocapsid protein (NP), and movement protein (MP). Subsequently, three additional segments (RNA5–RNA7), referred to as auxiliary segments, were identified, bringing the total genome to seven segments [7]. The functions of these auxiliary segments remain largely uncharacterized. However, comparative analyses within the genus suggest that they may play roles in vector transmission, suppression of host gene silencing, or modulation of host range [8]. Furthermore, it has been proposed that RNA5 and RNA7 of RRV may represent closely related homologs arising from a gene duplication event [9].

RRV is transmitted through grafting, whereby infected scions are joined to healthy rootstock and vice versa [10,11], as well as by the eriophyid mite vector, *Phyllocoptes fructiphilus* [2,12]. Eriophyid mites (Class: *Arachnida*, Subclass: *Acari*, family: *Eriophyidae*) belong to the superfamily *Eriophyoidea*, which includes approximately 5000 species [13]. These mites are commonly found in temperate regions, are characterized by having two pairs of legs, and are phytophagous in nature [13]. *P. fructiphilus* is a soft-bodied mite measuring approximately 140–175 μM in length [14]. Experimental studies have demonstrated that mechanical transmission of RRV via crude sap or infectious clones can occur in several non-rose hosts, including pepper, squash, tomato, pea, and zucchini. However, the establishment of infection in these hosts is transient and not sustained [15]. To date, members of the genus *Rosa* remain the only confirmed natural host of RRV [16].

Previous studies have suggested that the genetic diversity of RRV is relatively low, although these conclusions have largely been based on geographically limited datasets and may not fully represent the extent of variation within the species [1,9,17,18]. Therefore, additional whole-genome characterization of isolates from underrepresented geographic regions is needed to better understand RRV population structure and evolution. The objectives of this study were to (i) generate and characterize near-complete genome sequences of RRV isolates collected primarily in Oklahoma, (ii) determine their phylogenetic relationships relative to previously characterized isolates, and (iii) evaluate patterns of genetic diversity, population differentiation, and molecular evolution within RRV.

## 2. Materials and Methods

### 2.1. Design and Optimization of Primers Targeting the RRV Genome

Overlapping primer sets were designed to amplify the full-length coding sequences of RRV as amplification of the 3′ and 5′ terminal regions proved challenging (Table 1). Primers were designed using more than 100 publicly available RRV sequences retrieved from NCBI on 5 January 2026. Multiple sequence alignments were generated using MEGA version 11 [19], and primers were selected to target regions exhibiting high sequence conservation. All primers were designed using PrimerStat v2 and were initially optimized using gradient PCR [20].

### 2.2. Sample Collection and Nucleic Acid Isolation

Leaf samples from rose plants exhibiting characteristic symptoms of RRD were collected from garden nurseries, retail outlets, and natural sources throughout Oklahoma. Sampling locations, collection dates, and host symptoms were recorded for each sample. In addition, a subset of asymptomatic plants was included, given the potential for latent RRV infections [22]. Total RNA was extracted individually from each sample using the Norgen Plant RNA kit according to the manufacturer’s protocol (Norgen Biotek, Thorold, ON, Canada). RNA was eluted in 35 µL of nuclease-free water and assessed for purity and concentration using a Nanodrop 8000 spectrophotometer (ThermoFisher, Waltham, MA, USA). All samples were subsequently normalized to a working concentration of 300–400 ng/µL.

### 2.3. Screening by Reverse Transcription Polymerase Chain Reaction (RT-PCR)

Total RNA from each sample was reverse transcribed using SuperScript III reverse transcriptase (ThermoFisher, Waltham, MA, USA) following the manufacturer’s protocol with minor modifications. Briefly, 2–5 µL of total RNA was combined with random hexamer primers to prepare the initial Primer-RNA mixture, incubated at 65 °C for 5 min, and then chilled on ice for 2 min. The reverse-transcription reaction mixture was then added, and the reaction was incubated at room temperature for 5 min, followed by 55 °C for 1 h and 70 °C for 15 min.

Each rose sample was initially screened using the NADH dehydrogenase primer pair to assess RNA quality as previously described [21]. Subsequently, all samples were individually tested for RRV by RT-PCR using virus-specific primers (see Table 1). Amplified PCR products were visualized on 1% TBE agarose gel.

### 2.4. High Throughput Sequencing (HTS) and Bioinformatic Analysis Pipelines

Following RT-PCR screening, sixteen RRV-positive samples representing diverse regions in Oklahoma were selected for RNA-Seq analysis. RNA quality was assessed using Qubit and TapeStation, and libraries were prepared using the ZymoSeq RiboFree Total RNA Library Kit (Zymo Research, Irvine, CA, USA). Sequencing was performed on the Illumina NextSeq2000 (100 bp reads) (Illumina, San Diego, CA, USA) at the Oklahoma State University Genomics Facility (Stillwater, OK, USA).

Raw sequencing reads were assessed using FastQC v0.12.1 [23] and quality trimmed using Trimmomatic v0.40 [24]. Filtered reads were mapped against the *Rosa chinensis* reference genome (GCA_002994745.2) using HISAT2 v2.2.1 [25], and mapped host reads were removed using SAMtools v1.23 [26]. Remaining reads were assembled de novo using rnaSPAdes v4.2 [27]. Assembled contigs were screened for viral sequences using BLASTn v2.17, DIAMOND v2.2.8, and Kraken2 v2.17.1 [28,29,30]. Downstream data processing and visualization was conducted in R v4.6 using common packages such as Tidyverse v2.0 [31] and ggplot2 v4.0 [32]. All HTS-related bioinformatic analyses were conducted using the OU supercomputing system (OSCER, Norman, OK, USA).

### 2.5. Sanger Sequencing and Virus Assembly

One representative RRV isolate (R11) was amplified by RT-PCR using the overlapping primers listed in Table 1. Each PCR amplicon was subsequently purified using the Omega Cycle Pure PCR Purification Kit (Omega BIO-TEK, Norcross, GA, USA) and subjected to bidirectional Sanger sequencing through Eurofins Genomic (Louisville, KY, USA). Resulting sequencing reads were aligned to the closest related RRV reference sequences from NCBI using the “map Sanger reads to reference” function in Unipro UGENE v52 [33]. Base calls were manually curated, and ambiguous nucleotides were resolved based on manual chromatogram peak inspection in UGENE.

### 2.6. Maximum Likelihood Phylogenetic Inference

Maximum likelihood (ML) analyses were performed to evaluate the phylogenetic relationships among the 17 Oklahoma RRV isolates sequenced in this study and more than 100 publicly available isolates from the NCBI database (accessed: 1 May 2026). Available isolates containing complete coding sequences were included in the phylogenetic analyses. The isolates characterized from India (accession numbers KT223518.1 and KX013764.1) were excluded because the available sequences were shorter than 400 nt. Multiple sequence alignments were conducted using MEGA v11 [19], as well as the MSA v1.45.6 [34] and DECIPHER v3.8 [35] packages in R. Optimal nucleotide and amino acid substitution models were selected based on the lowest Bayesian Information Criterion (BIC) scores using MEGA and phangorn v2.12.1 [36]. Differences in the preferred nucleotide substitution models reflect variation in nucleotide frequencies, substitution rates, and overall model complexity. ML trees were inferred and visualized using phangorn and ggtree v4.2.0 [37], respectively, with 2000 bootstrap replicates to assess node support. Phylogenetic analyses were performed using (i) complete nucleotide sequences (NT), (ii) nucleotide coding sequences (CDS) and (iii) amino acid sequences (AA). Each genomic segment of RRV was analyzed independently, resulting in 23 phylogenetic trees. In addition, a concatenated dataset comprising CDS regions from all segments was constructed to provide a phylogenomic framework. No topological incongruence was observed among NT, CDS, and AA-based trees. Furthermore, rooted phylogenetic trees generated using *Emaravirus kiwii* sequences as the outgroup exhibited topologies consistent with those of the unrooted trees. Accordingly, unrooted, NT-based phylograms are presented and discussed throughout, alongside the concatenated CDS tree.

### 2.7. Bayesian Phylogenetic Inference

In addition to ML inference, Bayesian inference (BI) was conducted to estimate node and root ages of the RRV isolates, and maximum clade credibility (MCC) trees were generated for temporal interpretation. All BI analyses were performed using BEAST v2.7.7 [38], with the input file (.xml) created in BEAUti v2.7.7. Sampling dates (tip dates) were assigned based on the date of collection for all RRV isolates. The nucleotide substitution model (site model) was selected according to the lowest BIC scores obtained in MEGA. A strict molecular clock (mean clock rate—1.0) and a coalescent constant population prior were used. The Markov chain Monte Carlo (MCMC) chain length was set to 100,000,000 iterations. A total of 100,000 (10% burn-in) trees were sampled and analyzed in Tracer version 1.7.2 [39] to evaluate effective sample size (ESS). Root and node ages were estimated using the “tree.height” and “node ages” parameters in Tracer and FigTree v1.4.4 [40], respectively. MCC trees were generated using TreeAnnotator v2.7. Comparison of ML and MCC tree topologies revealed no significant inconsistencies; therefore, ML trees were used to represent phylogenetic relationships, while BEAST-derived estimates were used to infer root and node ages.

### 2.8. Evaluating Gene Flow and Genetic Structure

RRV groups or populations were defined based on clustering patterns observed in the ML phylogenetic tree. To investigate gene flow and population structure, we conducted analyses of molecular variance (AMOVA), principal coordinate analyses (PCoA), and calculated fixation indices (Fst). AMOVA was performed using the R package pegas v1.4 [41], following the hierarchical framework described by Excoffier et al. 1992 [42]. High among-population variance indicates stronger genetic structuring, whereas lower variance suggests greater mixing and gene flow among populations. PCoA (classical scaling) was conducted using the R package ape v5.8 to visualize genetic relationships among isolates [43]. Genetic differentiation was further quantified using Fst estimates. Pairwise Fst values were calculated using the Hudson method implemented in R, which is based on nucleotide diversity measures [44]. In addition, Fst was estimated using the Weir and Cockerham method as implemented in the R package hierfstat v0.5, using haploid SNP data derived from sequence alignments [45,46]. Fst values range from 0, indicating no genetic difference, to 1, indicating complete population differentiation. Statistical significance was assessed at α = 0.05.

### 2.9. Molecular Variation of RRV Isolates

All RRV isolates were analyzed using both MEGA 12.1 and R 4.5.3 programming to calculate pairwise genetic distance, nucleotide diversity (π), the number of polymorphic sites, Tajima’s D statistic, and dN/dS (nonsynonymous/synonymous) ratios.

Pairwise distances were calculated for each genomic segment and sequence type including complete nucleotide sequences (NT), nucleotide coding sequences (CDS), and amino acid sequences (AA). Nucleotide diversity (π) was estimated as the average pairwise difference among sequences, with lower values indicating reduced variation and higher values reflecting greater divergence (e.g., 0 = no variation; 0.100 = high divergence) [47]. The Genetic polymorphism was quantified as the proportion of polymorphic sites (PPS), calculated by dividing the number of segregating sites by the total number of sites to enable standardized comparisons across segments.

The Tajima’s D statistic (D) was used to assess whether the RRV population is evolving under neutral or non-neutral conditions. Values of D ≈ 0 indicate neutral evolution, whereas negative values (D < 0) suggest an excess of rare alleles consistent with population expansion, or purifying selection, and positive values (D > 0) indicate a deficit of rare alleles consistent with balancing selection or population contraction. Values below −2 or above 2 are generally considered strong evidence of non-neutral evolution.

dN/dS ratios were estimated using MEGA to evaluate selection pressures acting on individual genes. In addition, the R package seqinr v 4.2 [48] was used to analyze coding regions and identify genes under positive or purifying selection. In general, dN/dS > 1, =1, and <1 indicate positive, neutral, and purifying selection, respectively. Statistical significance for all analyses was assessed at α = 0.05.

### 2.10. Potential Recombination Events

Each genomic segment of RRV was analyzed for potential recombination events using Datamonkey’s online web-based platform [49,50]. Sequence alignment files were submitted to GARD (Genetic Algorithm for Recombination Detection) with the ‘run mode’ set to ‘normal’, while all other parameters were retained at their default settings. Recombination within RRV genomic regions was inferred based on the identification of recombination breakpoints and the presence of topological incongruences detected by GARD.

## 3. Results

### 3.1. Symptoms Associated with RRD

A total of 309 rose samples were collected from multiple counties across the state (Figure 1A), with 28% originating from Tulsa County. Fewer than 10 additional samples were obtained from neighboring states including Arkansas and Texas. During surveys, we observed typical RRD-like symptoms including rosette, reddening, excessive thorn proliferation, leaf deformation, and dieback (Figure 2).

### 3.2. RT-PCR, Sanger Sequencing, and Illumina-HTS

Overall, 87 samples (28%) tested positive for RRV by RT-PCR. From these, 17 samples were selected to represent diverse geographical regions within Oklahoma for complete genome sequencing (Table 2). The majority of these samples (*n* = 16) originated from multiple counties in Oklahoma (Figure 1B), while one sample was collected from Lubbock, TX. One isolate (R11) was fully sequenced using the Sanger method; however, due to its labor-intensive and costly nature, HTS was employed for the remaining isolates. Sixteen isolates were sequenced using Illumina-HTS generating approximately 12.8 to 22 million reads per sample. Of these reads, 32–87% mapped to the *Rosa chinensis* reference genome (accession: GCA_002994745.2) and subsequent assembly produced 43,000–333,000 contigs.

Most isolates sequenced in this study contained all RNA segments (RNA1–RNA7). RNA7 was not recovered from isolates R115, R157, and R163 by HTS, while R91 and R114 contained divergent sequences associated with RNA2 and RNA5, respectively, and RNA1 from R156 was recovered only as fragmented sequence data (Table S1). Such variation is consistent with previous reports, in which similar observations have been documented [47,51].

### 3.3. Phylogenetic Analysis of RRV Isolates

Maximum-likelihood (ML) analysis of the concatenated coding-sequence dataset revealed two major groups: Group 1, designated as the OK Clade, and Group 2, which comprised the remaining RRV isolates. Group 1 consisted of eight isolates, including seven isolates identified in this study and one previously reported Oklahoma isolate available in GenBank (Figure 3A). Population differentiation analysis demonstrated substantial genetic divergence between these groups, as indicated by a high fixation index (Fst = 0.95) (Table 3). Analysis by AMOVA showed that 64.8% of the genetic variation was attributable to differences among-populations (ΦST = 0.64, *p* = 0.0001), indicating strong genetic differentiation and population structure between the two groups (Figure 3A). The PCoA analysis further supported this separation by revealing distinct clustering patterns, with the OK Clade positioned separately and perpendicular to the remaining isolates. PCoA1 explained 96.5% of the observed variance (Figure 3A). Collectively, these findings indicate substantial genetic differences between Group 1 (OK Clade) and Group 2 RRV isolates.

ML phylogenetic analyses of individual segments revealed patterns that were largely consistent with those observed using the concatenated CDS dataset. Specifically, RNA1, RNA2, RNA3, RNA5, and RNA6 maintained the OK Clade structure observed in the concatenated CDS analysis, consisting of eight RRV isolates (R11, R42, R100, R114, R163, R195, R204, and OK-1) (Figure 3). These phylogenetic relationships were further supported by population genetic analysis statistics including Fst, AMOVA, and PCoA (Table 3 and Figure 3). Collectively, these analyses demonstrate substantial genetic differentiation between the defined populations, Group 1 and Group 2.

In contrast, RNA4 exhibited a unique phylogenetic topology (Figure 3E). Only R100, R163, R195, and R204 clustered together to maintain the OK Clade, whereas the remaining Oklahoma isolates previously associated with this clade were distributed throughout the phylogram. These isolates are grouped more closely with other isolates originating from other geographic regions including Arkansas, Kentucky, California, etc. Although the pairwise Fst estimate remained high (Fst = 0.92), AMOVA attributed only 17.1% of the total genetic variation to differences between the defined groups, and this component was not statistically significant (ΦST = 0.17, *p* = 0.1149) (Figure 3E). These results suggest less genetic differentiation among the defined populations. Similarly, PCoA revealed distinguishable but overlapping clusters, with the OK Clade isolates embedded within the larger Group 2 cluster (colored green). The PCoA1 analysis showed 87.8% of the observed variance (Figure 3E). Together with the overlapping PCoA clusters, these results indicate weaker and less consistent population structure for RNA4 than for several other genomic segments.

Analysis of RNA7 revealed an additional reduction in the OK Clade, which consisted of isolates R11, R100, R114, R195, R204 and OK-1 (MZ703634.1) (Figure 3H). Population differentiation analysis indicated differences between these groups (Fst = 0.88) (Table 3). However, AMOVA demonstrated that only 9.4% of the genetic variation was explained by among-population differences (ΦST = 0.094, *p* = 0.0294) (Figure 3H), indicating substantially weaker population differentiation for RNA7 than observed for several other genomic segments (Figure 3H). The PCoA further supported these findings by revealing overlapping clusters, with the OK Clade encompassing many of the remaining isolates. The PCoA1 accounted for 86.9% of the total variation (Figure 3H). Overall, these analyses suggest that although genetic differences between the OK Clade and Group 2 persist within RNA7, the extent of divergence is lower than that observed for other RRV segments particularly RNA1 and RNA6.

A second geographically associated pattern was observed for a well-supported subclade provisionally designated the TX Clade. This clade included isolates R84 and R196 characterized in the present study together with previously reported Texas isolates. Importantly, all Texas-derived isolates consistently grouped together to form the TX Clade. In contrast, the remaining isolates characterized in this study generally clustered with isolates from multiple geographic regions across the US with no clear geographic patterns observed.

### 3.4. Assessing Root Age and Divergence via BEAST

The mean root ages and corresponding 95% highest probability density (HPD) intervals were estimated for each genomic segment and the concatenated genome dataset (Figure 4 and Table S2). Root age estimates represent the inferred divergence time between Group 1 (OK Clade) and the remaining RRV isolates comprising Group 2. For example, the estimated mean root age for the concatenated genome was 138.56 years (Figure 4). Overall, most mean age estimates exhibited 95% HPD intervals overlapping around approximately 380 years (indicated by the horizontal yellow line in Figure 4). Based on these evolutionary models, the divergence of the OK Clade from Group 2 isolates likely occurred more than 100 years ago, with the most plausible divergence timeframe estimated between 150 and 450 years ago. Additionally, divergence time estimates were generated for the TX clade, which indicated a more recent origin with an estimated time of approximately 15–25 years from its most recent common ancestor. Collectively, these findings suggest that the OK Clade represents an older divergence event within the RRV species, whereas the TX clade reflects a comparatively recent diversification event.

### 3.5. Pairwise Distance, Nucleotide Diversity, and Proportion of Polymorphic Sites

Isolates within the OK Clade, together with R196 from Group 2, contained sequence variation predicted to disrupt the RNA6a open reading frame. The predicted ORF disruption was identified using NCBI ORF Finder and was previously noted for isolate NVWA6166361/OK-1 (MZ703633.1). Because the functional consequence of these mutations has not been experimentally validated, RNA6a was excluded from subsequent amino-acid-level comparative analyses.

Analysis of complete nucleotide sequences (NT) revealed pairwise distances ranging 0.00014–0.13. Among genomic segments, RNA3 and RNA4 exhibited the lowest nucleotide diversity (π= 0.014 and 0.015, respectively), whereas segments RNA1 and RNA5 displayed the highest nucleotide diversity (π = 0.022 and 0.030, respectively) (Table 4). The proportion of polymorphic sites (PPS) ranged from 0.20–0.34 across segments (Table 4). RNA7 exhibited the lowest PPS value (PPS = 0.20), whereas RNA3 displayed the highest (PPS = 0.34) (Table 4).

The CDS analyses revealed pairwise distances ranging from 0.00015–0.154. The lowest nucleotide diversity values were observed for RNA3 and RNA6b, whereas RNA5 and RNA6a exhibited the highest nucleotide diversity (π = 0.030 and 0.022, respectively). The PPS estimates for CDS datasets ranged from 0.14–0.25 (Table 4). RNA6b and RNA3 were proportionally the least polymorphic genes (PPS = 0.14 and 0.16, respectively), whereas RNA6a and RNA4 displayed the highest level of polymorphism (PPS = 0.25 and 0.22, respectively).

Pairwise distances calculated from amino acid sequences ranged from 0.00021–0.096. Proteins encoded by RNA1 and RNA4 exhibited the lowest average pairwise distances (0.004 and 0.006, respectively), whereas proteins encoded by RNA5 and RNA7 displayed the highest average distances (0.022 and 0.015, respectively) (Table 4). RNA1 and RNA7 exhibited the lowest amino acid-level PPS values (PPS = 0.07 and 0.13, respectively). whereas proteins encoded by RNA4 and RNA2 displayed the highest levels of polymorphism (PPS = 0.27 and 0.24, respectively).

### 3.6. Tajima’s D Statistic

Analysis of Tajima’s D values across complete nucleotide sequences of RRV revealed significant negative values (D values −2 or less) for RNA2, RNA3, RNA4, and RNA6, suggesting an excess of low-frequency polymorphism and the presence of rare alleles for these segments. RNA1, RNA5, and RNA7 also exhibited negative Tajima’s D values; however, these values were weaker and not statistically significant suggesting less pronounced deviations from neutrality (Table 4).

Evaluation of Tajima’s D for nucleotide coding sequences (CDS) revealed that RNA4 exhibited a Tajima’s D value below −2, indicating a strong excess of rare variants and significant deviation from neutral expectations. The coding sequence of RNA1, RNA2, RNA3, RNA6a, RNA6b, and RNA7 exhibited negative Tajima’s D values ranging between −1 and −2; however, these values were not statistically significant, suggesting weaker evidence for an excess of low-frequency alleles. Among the coding sequences evaluated, RNA5 exhibited the Tajima’s D value closest to neutrality (Tajima’s D = −0.77). The concatenated coding sequence dataset of RRV produced a significant Tajima’s D value of −2.41 (*p* < 0.05), indicating a significant excess of rare alleles and supporting deviations from neutral evolutionary expectations. These negative Tajima’s D values indicate an excess of low-frequency variants relative to neutral expectations; however, the underlying causes may include demographic processes, population structure, or selection and cannot be distinguished from Tajima’s D alone.

### 3.7. Nonsynonymous (dN) and Synonymous (dS) Mutations

Selection pressures acting on RRV coding sequences were evaluated using the codon-based z test implemented in MEGA v.11 with 1000 bootstrap replicates. All individual RRV genes as well as the concatenated genome dataset exhibited evidence of negative (purifying) selection. To further characterize these relationships, dN/dS patterns were explored and visualized using R programming. Mean dN/dS ratios were calculated for each coding sequence to assess the relative strength of selective constraint across genomic segments. Among all genes analyzed, RNA7 exhibited the highest mean dN/dS ratio (dN/dS = 0.33 ± 0.004), whereas RNA1 exhibited the lowest mean ratio (dN/dS = 0.071 ± 0.001) (Figure 5). Despite variation in dN/dS magnitude among genomic segments, mean dN/dS ratios for all coding regions were below 1, consistent with an overall predominance of purifying selection across the RRV genome.

### 3.8. Recombination Analysis

Potential recombination events within the RRV genome were investigated using GARD. Several putative recombination breakpoints were identified, resulting in alternative phylogenetic topologies and favoring multiple-tree models for specific genomic segments. For RNA1, GARD identified potential recombination breakpoints at nucleotide positions 4991 and 5000. The multiple-tree model was favored over the single-tree model, suggesting that at least one of these inferred breakpoints may represent genuine topological incongruence within the dataset.

Similarly, RNA5, contained two inferred recombination breakpoints located at nucleotide positions 707 and 747, with the multiple-tree model again providing a better fit, suggesting potential phylogenetic incongruence associated with at least one breakpoint. For the remaining genomic segments, GARD detected no evidence supporting recombination events, and the multiple-tree models were not favored over the single tree model. GARD identified potential recombination-associated incongruence in RNA1 and RNA5, whereas no breakpoint support was detected for the remaining genomic segments.

## 4. Discussion

This study expands the known genomic diversity of RRV through characterization of 17 isolates collected primarily in Oklahoma. The major finding was the identification of a geographically associated lineage, designated the OK Clade, which was consistently differentiated from most previously characterized RRV isolates across the concatenated genome and individual RNA segments. These findings demonstrate that the population structure of RRV is more complex than previously recognized.

ML phylogenetic analyses generated largely consistent topologies when genomic segments were evaluated individually or as concatenated datasets. For most analyses, the GTR + G + I nucleotide substitution model was selected as the best-fitting model. In contrast, the HKY + G + I model was preferred for RNA6 and RNA7. A previously unrecognized lineage in the datasets examined here, designated as the OK Clade (i.e., Group 1), was consistently observed. This clade typically contained seven isolates characterized in this study (R11, R42, R100, R114, R163, R195, and R204) together with the previously reported isolate NVWA6166361/OK-1. Examination of isolate origin revealed clear geographical clustering as all isolates comprising the OK clade originated from roses collected in Oklahoma, with most isolates localized to the northeastern portion of the state (Figure 6). Previous studies did not identify this lineage because earlier analyses predated the availability of the OK-1 isolate sequence or questioned the reliability of its sequence data [9,17]. The inclusion of newly characterized isolates allowed for a more robust assessment of the evolutionary placement of this lineage.

A second geographically associated lineage, provisionally designated as the TX Clade, was also consistently observed via various analyses. Previous studies [9,17] had inferred clustering among several Texas-derived RRV isolates, and our analyses have further expanded these lineages through the inclusion of isolates R84 and R196. Isolate R84 originated from Love County located in southern Oklahoma near the Texas–Oklahoma state line, whereas R196 was collected in Lubbock, Texas. Apart from the OK and TX clades, no additional geographical associations were observed. Although not shown here, an additional phylogenetic analysis was performed that included the two available isolates from India (accession numbers KT223518.1 and KX013764.1). Both isolates clustered within Group 2 and did not form a distinct clade. Rooted phylogenetic analyses using *Emaravirus kiwii* as the outgroup also provided further insight into the evolutionary relationships among RRV isolates. Under the models and analytical methods used, the OK Clade occupied a basal position relative to the remaining RRV isolates. This placement suggests that the Oklahoma isolates comprising the OK Clade may represent an early-diverging lineage within RRV. Previous authors proposed that movement of infected plant material, recent emergence of RRV within cultivated roses, and relatively slow evolutionary rates may contribute to the absence of strong geographical structure for RRV [17].

Population genetic analyses further supported differentiation between the OK Clade and the remaining isolates (Group 2). AMOVA analyses indicated patterns consistent with restricted gene flow, suggesting genetic differentiation. The comparatively high genetic differentiation between the OK Clade and Group 2 is consistent with substantial population structure. Lower diversity within the OK Clade relative to the remaining isolates may reflect historical demographic processes, including founder effects or population bottlenecks; however, additional sampling and demographic modeling will be required to distinguish between these possibilities. Although the capacity of RRV to replicate within its primary vector remains unclear, vector-associated bottlenecks may contribute to these evolutionary patterns [1].

The PCoA analysis revealed substantial variation in genetic structure among genomic segments. RNA1, RNA6, and the concatenated genome analyses demonstrated clear separation between the OK Clade and remaining Group 2 isolates, whereas other genomic segments exhibited varying degrees of overlap. These results suggest that patterns of differentiation and gene flow are segment dependent and indicate that the defined populations may be evolving semi-independently with limited genetic differentiation.

The segment-dependent changes in phylogenetic placement observed for several isolates may also warrant investigation in the context of reassortment. Because RRV possesses a segmented genome, exchange of genomic segments among coinfecting viral variants could contribute to incongruent phylogenetic relationships among individual RNA segments. Although the present analyses do not establish reassortment, comparison of segment-specific topologies suggests that this evolutionary mechanism should be examined in future studies.

Root age estimates further suggest that divergence between Group 2 isolates and the OK Clade occurred relatively early during the evolutionary history of RRV. Mean divergence estimates and 95% highest posterior density intervals suggest lineage splitting approximately 150–450 years ago. In contrast, the TX Clade exhibited a considerably younger estimated divergence time of approximately 15–25 years. Combined ML and Bayesian analyses therefore suggest a prolonged mostly independent evolution of the OK Clade.

Despite these observations, isolates within the OK Clade do not satisfy current species demarcation criteria (at least two of RNA1–RNA4 gene products must differ by more than 20%) for emaraviruses and therefore remain taxonomically classified within the same viral species [6]. Nevertheless, phylogenetic reconstruction, population genetic analyses, and divergence time estimation collectively support recognition of the OK Clade as a distinct evolutionary lineage within the RRV species.

Compared with previous studies reporting relatively low genetic diversity within RRV populations [1,9,17], our analyses suggest that RRV diversity is greater than previously recognized. Other studies on emaraviruses have suggested that the nucleotide diversity can vary from relatively high [52,53,54] to low genetic diversity [55].

Overall mean pairwise distances increased relative to prior investigations, and nucleotide identity among isolates ranged from approximately 87–100%, substantially expanding previous estimates [17]. While RNA5 consistently exhibited elevated diversity across studies, RNA3 and RNA4 displayed comparatively lower variability. These findings collectively suggest greater genomic complexity and diversification within RRV populations than previously reported.

Neutrality analyses consistently produced negative Tajima’s D values, suggesting that RRV populations are evolving under non-neutral evolutionary processes. Possible explanations include population expansion, purifying selection, or selective sweeps. Similar conclusions have previously been made and are consistent with ongoing lineage diversification [9].

Analyses of dN/dS ratios demonstrated that gene-wide dN/dS patterns were consistent with an overall predominance of purifying selection. This pattern is unsurprising given that RRV must successfully replicate within both plant hosts and arthropod vectors, potentially imposing strong selective constraints [56]. Although RNA7 exhibited the highest dN/dS ratios among analyzed segments, all values remained consistent with purifying selection. Conversely, RNA1 exhibited the strongest evidence of evolutionary constraint, which is expected for genes encoding replication-associated functions [57].

The results of these analyses suggest that recombination has played only a limited role among the RRV isolates examined. Although potential recombination breakpoints were identified in RNA1 and RNA5, most genomic segments showed no evidence of recombination-associated breakpoints. These findings are broadly consistent with the relatively limited homologous recombination reported for negative-sense RNA viruses [58,59]. However, the candidate regions in RNA1 and RNA5 warrant further investigation because recombination can contribute to the generation of genetic diversity and influence virus evolution [60].

These findings suggest that the OK Clade represents an evolutionarily distinct lineage that has apparently coexisted with other RRV populations for an extended period while maintaining possible historical isolation and genetic differentiation. The underlying drivers responsible for divergence remain uncertain but may include historical bottlenecks, founder effects, geographic isolation, expanded host associations, or differences in transmission efficiency.

The comparatively conserved regions identified in RNA3 and RNA6 may represent candidates for future evaluation as molecular diagnostic targets. In contrast, the greater sequence variability observed in RNA5 suggests that assays targeting this segment may require particular attention to sequence diversity. These suggestions should be examined experimentally across geographically and genetically diverse RRV isolates.

## 5. Conclusions

This study expands the known genetic diversity of RRV and identifies a geographically associated lineage, designated the OK Clade, that is genetically differentiated from most previously characterized isolates. Phylogenetic and population genetic analyses consistently supported differentiation of this lineage, although the extent of separation varied among genomic segments. These segment-dependent patterns highlight the complexity of RRV evolution and warrant additional investigation of mechanisms such as recombination and reassortment.

The greater sequence diversity observed in this study compared with previous reports indicates that the genetic structure of RRV may be more complex than previously recognized. Broader geographic sampling, continued whole-genome sequencing, and experimental characterization of genetically distinct isolates will be necessary to determine the distribution, biological significance, and epidemiological consequences of the OK Clade and other potentially unrecognized RRV lineages. These findings provide a foundation for further studies of RRV evolution and for the continued development of robust diagnostic and disease-management strategies.

## Figures and Tables

**Figure 1:**
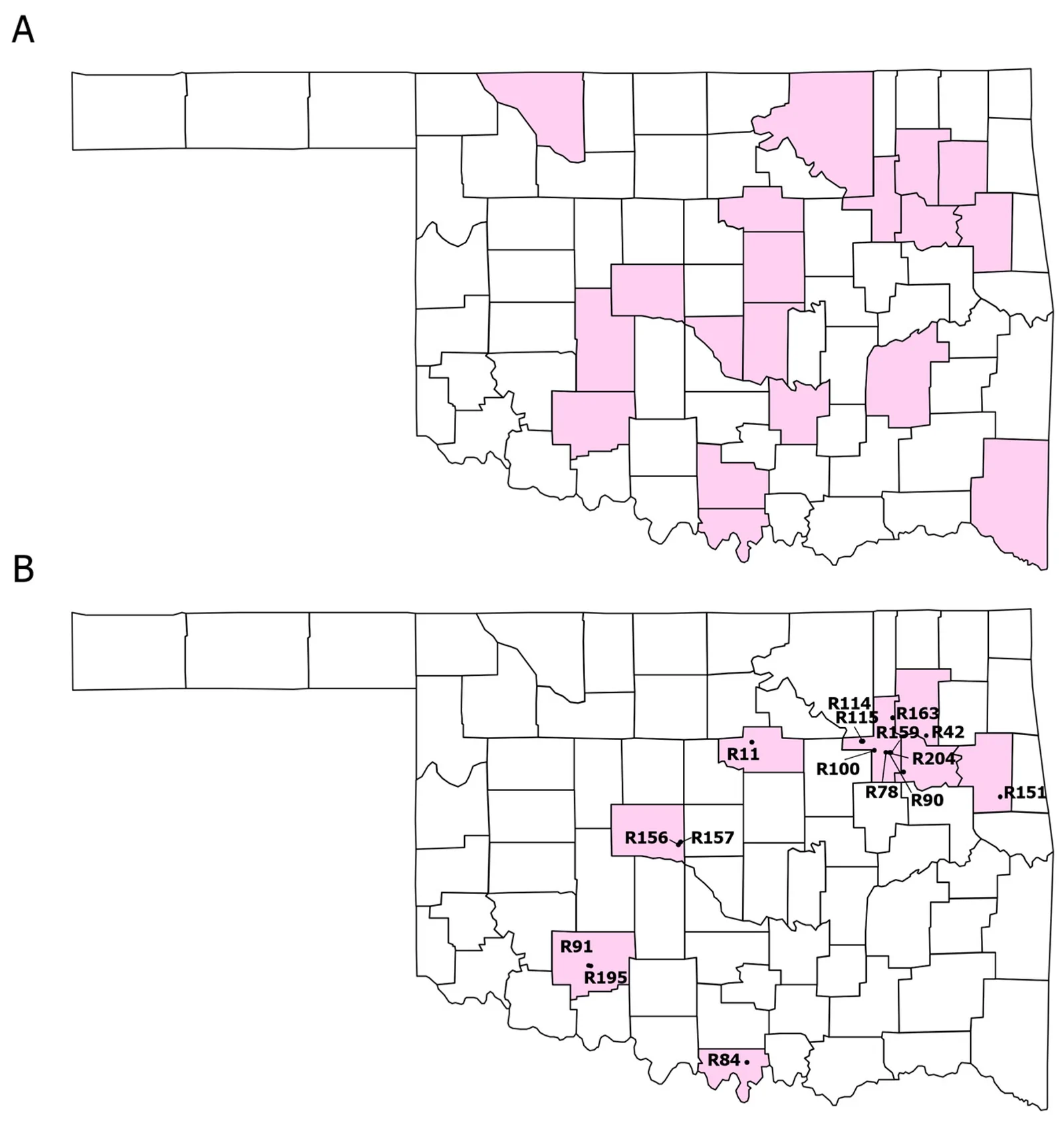
Simplified maps of Oklahoma counties. (**A**) Counties in which rose samples were collected are shaded in pink. (**B**) Counties from which RRV isolates sequenced in this study were obtained are shaded in pink.

**Figure 2:**
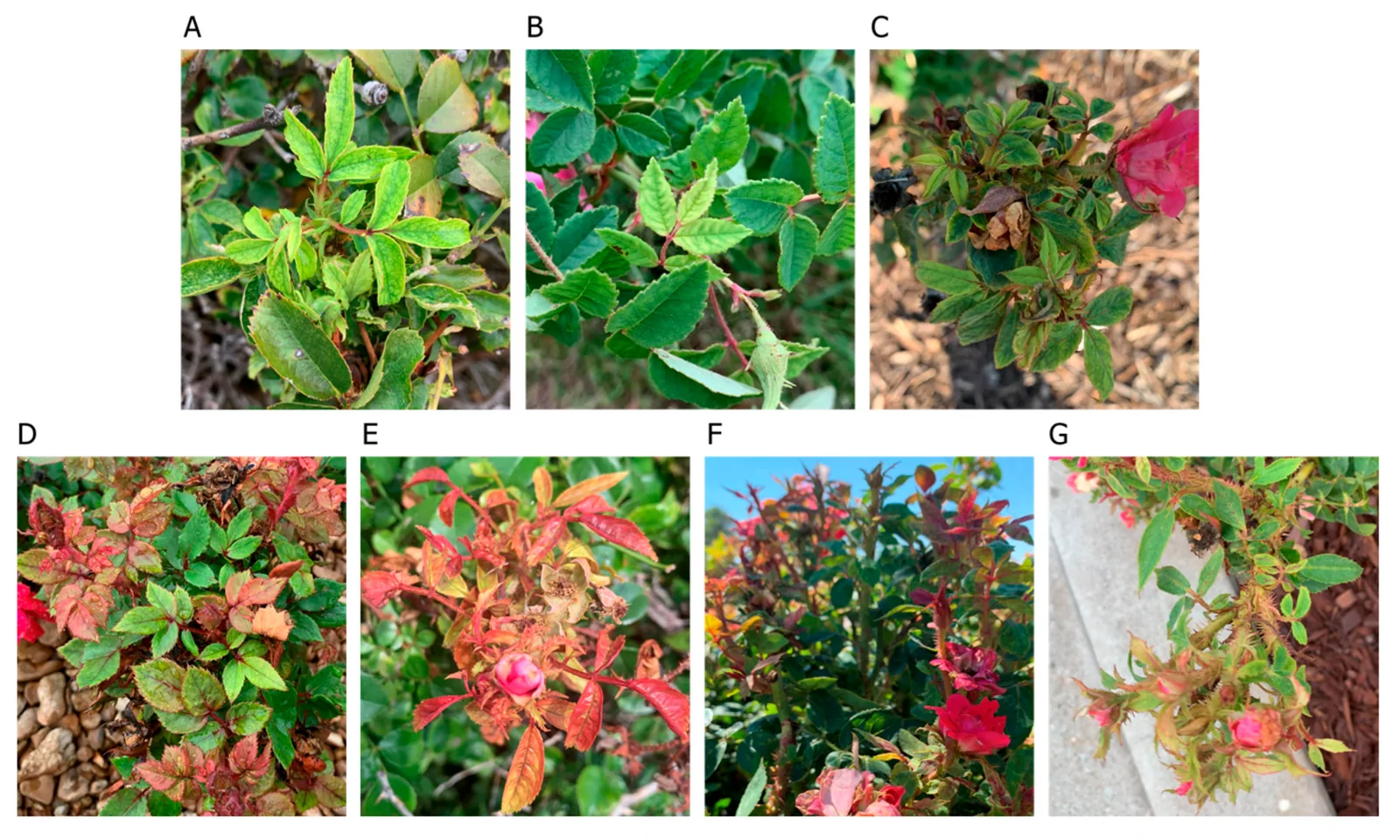
Symptoms associated with rose rosette disease (RRD). (**A**,**B**) Mosaic and vein banding of the leaves; (**C**) small, deformed leaves with vein banding; (**D**,**E**) prolonged reddening; and (**F**,**G**) excessive thorniness, leaf deformation, and vein banding.

**Figure 3:**
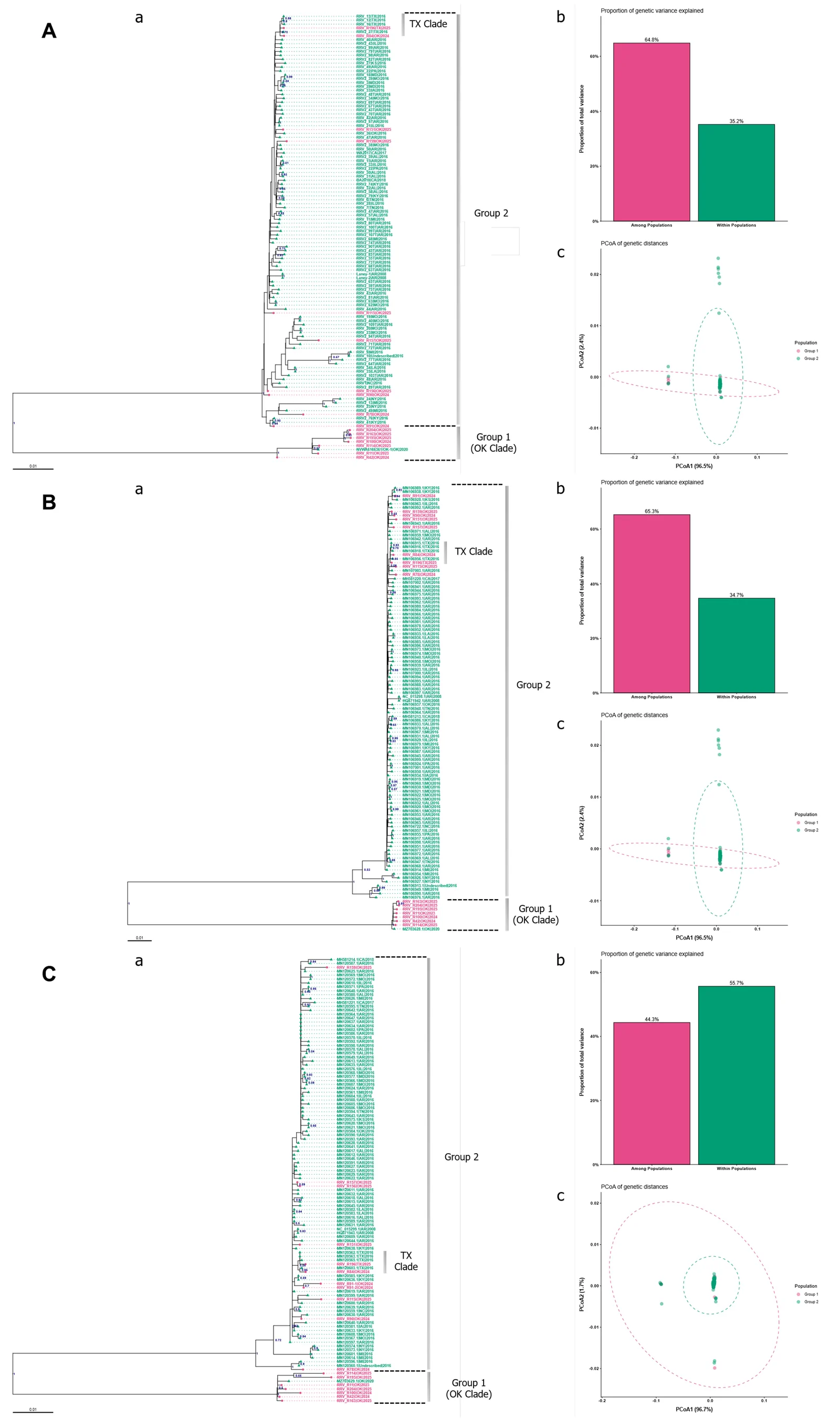

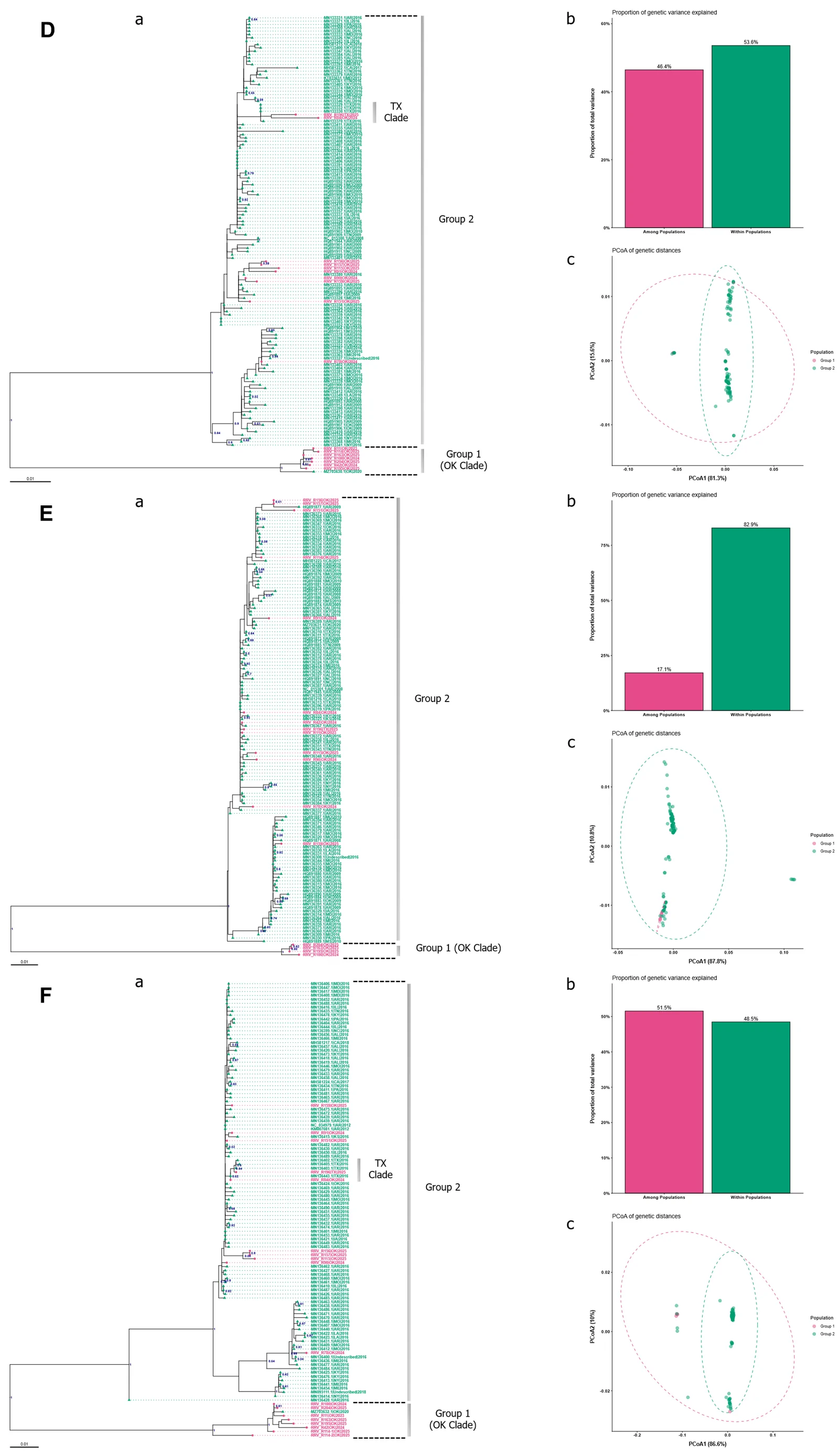

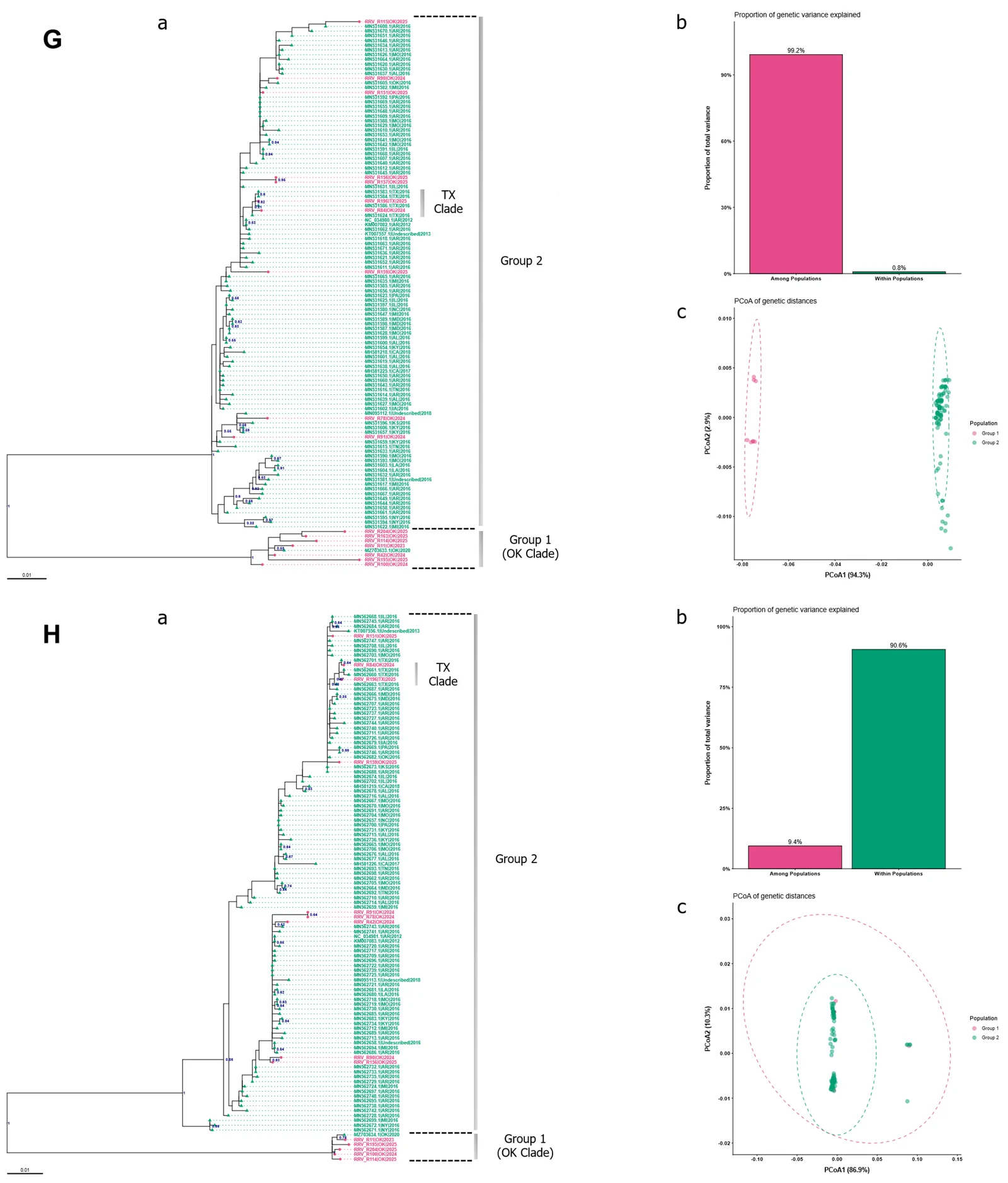
Phylogenetic and population genetic analyses of the concatenated coding sequence (CDS) and individual genomic segments of rose rosette virus (RRV). For each dataset, analyses include (**a**) maximum-likelihood (ML) phylogenetic tree, (**b**) analysis of molecular variance (AMOVA), and (**c**) principal coordinate analysis (PCoA) illustrating the separation of Group 1 and Group 2. Isolates sequenced in this study are highlighted in pink. (**A**) Concatenated CDS dataset (best-fit nucleotide substitution model: GTR + G + I; taxa = 113). (**B**) RNA1 complete nucleotide sequence (GTR + G + I; taxa = 113). (**C**) RNA2 complete nucleotide sequence (GTR + G + I; taxa = 114). (**D**) RNA3 complete nucleotide sequence (GTR + G + I; taxa = 137). (**E**) RNA4 complete nucleotide sequence (GTR + G + I; taxa = 137). (**F**) RNA5 complete nucleotide sequence (GTR + G + I; taxa = 116). (**G**) RNA6 complete nucleotide sequence (HKY + G + I; taxa = 116). (**H**) RNA7 complete nucleotide sequence (HKY + G + I; taxa = 113).

**Figure 4:**
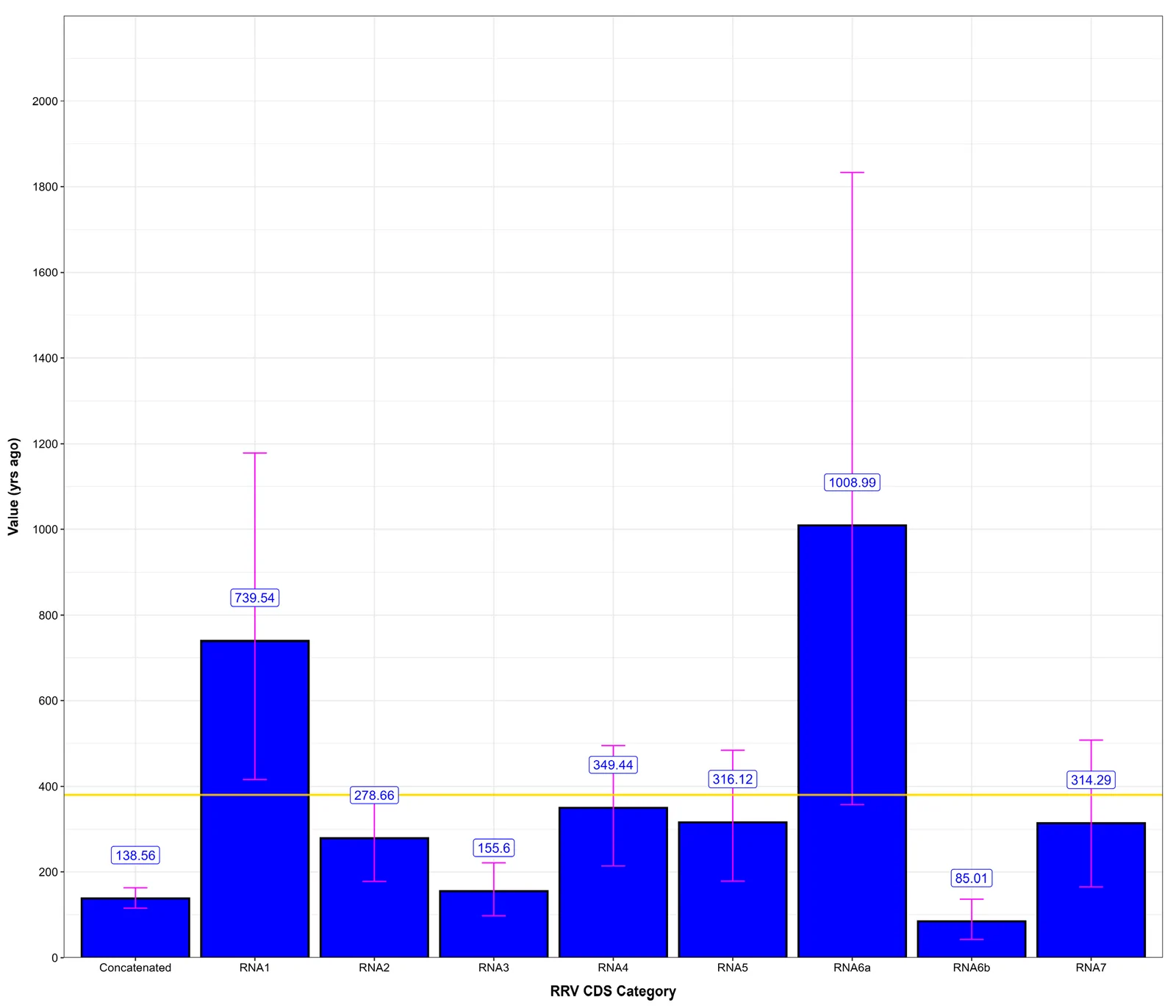
Root age estimates for the concatenated coding-sequence dataset and individual RRV genomic segments based on Bayesian phylogenetic analyses visualized using output from Tracer. Pink error bars indicate the 95% highest posterior density (HPD) intervals for each estimate. The yellow horizontal line denotes the approximate time point at which the majority of the 95% HPD intervals overlap.

**Figure 5:**
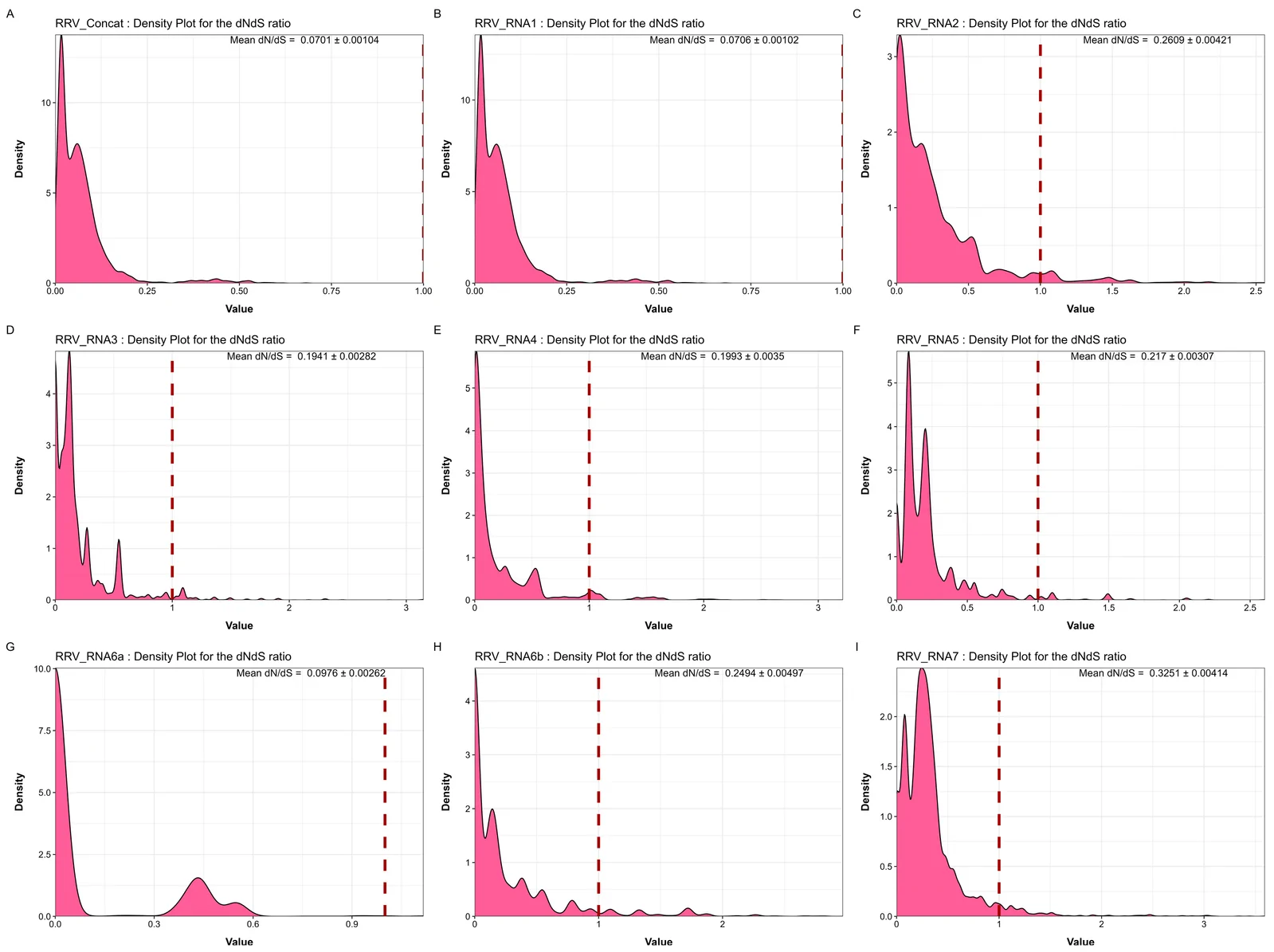
Density distributions of dN/dS ratios for the concatenated CDS and individual RRV coding regions. (**A**) concatenated CDS, (**B**) RNA1, (**C**) RNA2, (**D**) RNA3, (**E**) RNA4, (**F**) RNA5, (**G**) RNA6a, (**H**) RNA6b, and (**I**) RNA7. The red dashed line indicates a dN/dS ratio of 1, representing the expectation under neutral selection.

**Figure 6:**
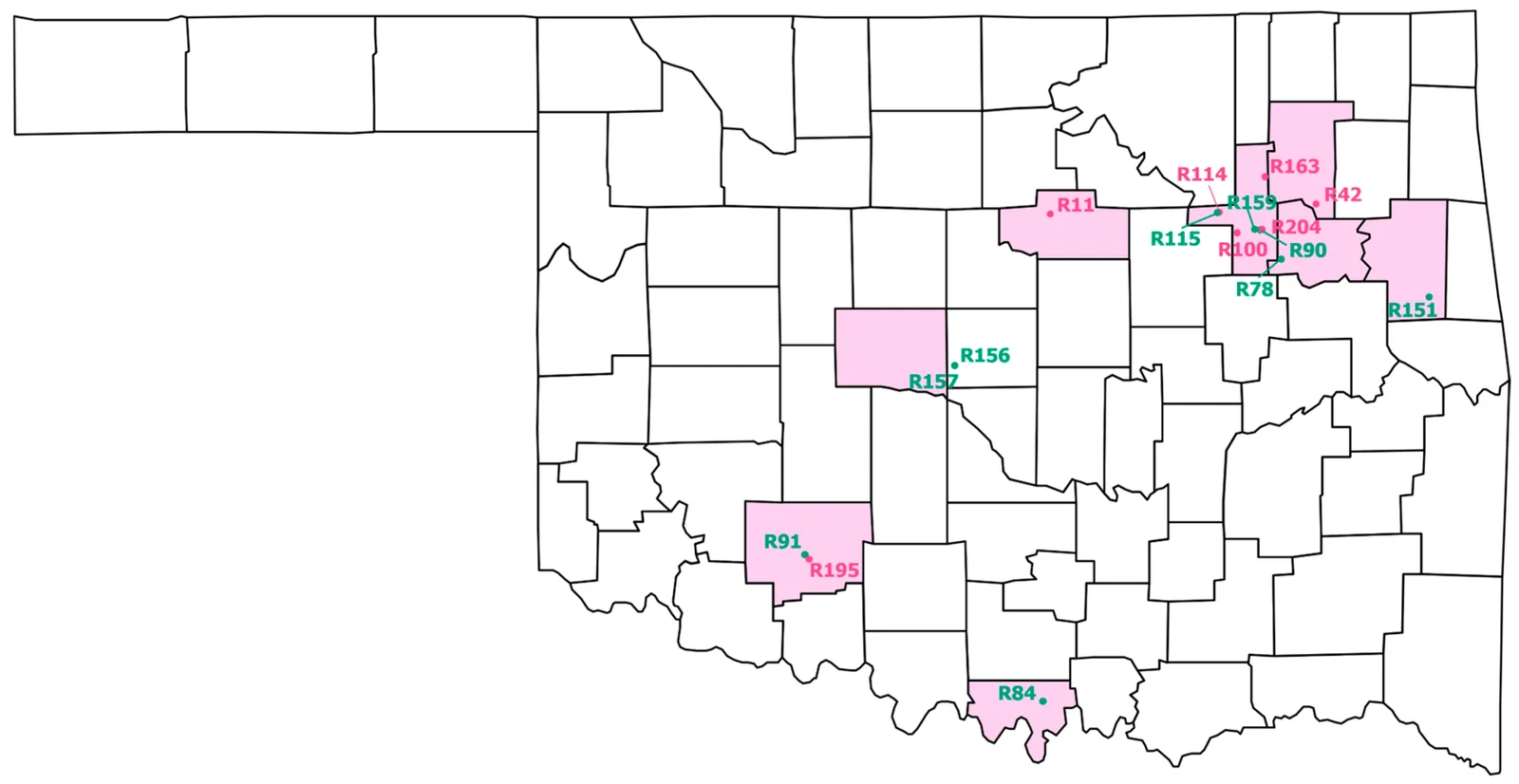
Map of Oklahoma counties showing the geographic distribution of RRV isolates sequenced in this study. Counties in which rose surveys were conducted are shaded in pink. Isolates shown in pink and green represent Group 1 and Group 2, respectively.

**Table 1:** Primers used for the detection and molecular characterization of rose rosette virus (RRV).

Primer Name	Sequence 5′-3′	Amplification Size	Target	Annealing Temp (C)	Reference
NADH ND-2F	GGACTCCTGACGTATACGAAGGATC	721	NADH	57	[21]
NADH ND-2R	AGTAGATGCTATCACACATACAAT				
RRV963-F	CTTGGAGTTCACATTAAAACAGC	963	RNA3	53	This study
RRV963-R	CTGATATTTATTGTGCACCTC				
RRV-F1	GGTTAATGCCAACAAATTCAGATGC	1086	RNA1	58	This study
RRV-R1	CTAATCTAGACAAAATCCAAA				
RRV-F2	CTAAAGTAGAAACTGTCACAATGGC	970	RNA1	53	This study
RRV-R2	CCTTGACACATGTAATATGCTAGTG				
RRV-F3	CCACTATAATCGTAGGGAATG	1049	RNA1	58	This study
RRV-R3	ATGAGGGTCTTGCCATTGTAAC				
RRV-R4	CCTGAATATGATGCTAAATCTTG	969	RNA1	53	This study
RRV-F4	CAATATACGAGAATGGTGAATGGT				
RRV-F5	CAACAACACTAGGACATGCATAC	985	RNA1	53	This study
RRV-R5	GTATAGATTTCCCGATCATCAGC				
RRV-F6	ATCTATTACTGACAGTGAAGAAGA	850	RNA1	58	This study
RRV-R6	GACTTAGATTGTTGTTAAAACCTG				
RRV-F7	CCAGATCGTATCAGTGATGTTAC	989	RNA1	53	This study
RRV-R7	GAGACTAGTTCATTGGCATAAG				
RRV-F8	TTCAAAAGCAATTATGGAAGC	792	RNA1	50	This study
RRV-R8	GCATGTTCAGAGACACTATTC				
RRV-F9	ATGGACAATTCTGTGATCAACC	1004	RNA2	58	This study
RRV-R9	ATCTCAGTTCAGCAGATGTTACTC				
RRV-F10	CTCCTCATAAAACTAGTCAAC	1257	RNA2	53	This study
RRV-R10	CCATAGGTGAATTCAGTACATAC				
RRV-F11	GTTGAACTTGCTAGAGACATTGG	609	RNA3	53	This study
RRV-R11	CCCATAATTTATCTAAGCTAACG				
RRV-F12	TCACTTTCCCTCTAAGTCTTCCA	766	RNA4	58	This study
RRV-R12	TAAGTTAAGTGATGGAATCTAGAC				
RRV-F13	CTGTGAGTGAGAACACCTAAGT	765	RNA4	58	This study
RRV-R13	CTTTCCTGACATCATTGAAACCTC				
RRV-F14	GATTGAGAGAAGTGAAAATAGAG	749	RNA5	58	This study
RRV-R14	CTAACCAAGTGGCCTAAATCAAC				
RRV-F15	ACTCTTTTAAAACTTGCACCAG	1087	RNA5		This study
RRV-R15	CATCTGAAATATTCTTCTTCATGG				
RRV-F16	GTGGTCCTAGGTTATGATCCAG	793	RNA6	50	This study
RRV-R16	GCAATATATCTTTGTTCAAATTCC				
RRV-F17	AAGCAAATCATATTTAGGTCAACC	597	RNA6	53	This study
RRV-R17	CAATCTGCCAGTGGTATAAGCAA				
RRV-F18	CCTTCTGAATCATGTTCTTCCAAG	910	RNA7	53	This study
RRV-18R	TTCATAGATGGCTAGCTTTTATTC				
RRV-F19	CTCCCACAATTAAATAGATTTCG	813	RNA7	53	This study
RRV-R19	GAGTCTGATTCAGAAATTGTAGTG				

**Table 2:** List of RRV isolates collected and sequenced in this study.

City	County	State	Isolate ID	Collection Date
Broken Arrow	Wagoner	Oklahoma	R78	30 July 2024
Broken Arrow	Tulsa	Oklahoma	R204	29 August 2025
Fort Gibson	Cherokee	Oklahoma	R151	2 June 2025
Inola	Rogers	Oklahoma	R42	20 April 2024
Lawton	Comanche	Oklahoma	R91	16 September 2024
Lawton	Comanche	Oklahoma	R195	4 August 2025
Lubbock	Lubbock	Texas	R196	16 August 2025
Marietta	Love	Oklahoma	R84	4 September 2024
Owasso	Rogers	Oklahoma	R163	10 July 2025
Sand Springs	Tulsa	Oklahoma	R114	24 April 2025
Sand Springs	Tulsa	Oklahoma	R115	1 May 2025
Stillwater	Payne	Oklahoma	R11	25 October 2023
Tulsa	Tulsa	Oklahoma	R90	6 September 2024
Tulsa	Tulsa	Oklahoma	R100	14 October 2024
Tulsa	Tulsa	Oklahoma	R159	25 June 2025
Yukon	Canadian	Oklahoma	R156	23 June 2025
Yukon	Canadian	Oklahoma	R157	23 June 2025

**Table 3:** Population differentiation estimates for the concatenated genome and individual genomic segments of rose rosette virus (RRV).

Analysis Type	Clade/Population	n ^1^	π ^2^	Within (π)	Between (π)	Total (π)	Fst
Concatenated	Group 1	8	0.0031	0.0059	0.1214	0.0223	0.9516
	Group 2	103	0.0061				
RNA1	Group 1	8	0.0031	0.0058	0.1249	0.0219	0.9536
	Group 2	105	0.0060				
RNA2	Group 1	8	0.0029	0.0044	0.0948	0.0170	0.9531
	Group 2	106	0.0046				
RNA3	Group 1	8	0.0030	0.0078	0.0779	0.0135	0.8994
	Group 2	129	0.0081				
RNA4	Group 1	4	0.0028	0.0090	0.1198	0.0151	0.9245
	Group 2	132	0.0092				
RNA5	Group 1	9	0.0106	0.0141	0.1244	0.0292	0.8869
	Group 2	107	0.0144				
RNA6	Group 1	8	0.0048	0.0063	0.0796	0.0161	0.9212
	Group 2	108	0.0064				
RNA7	Group 1	6	0.0037	0.0102	0.0911	0.0189	0.8880
	Group 2	107	0.0106				

Note: Group 1 represents the OK Clade, whereas Group 2 comprises all remaining RRV isolates. ^1^ number of isolates; ^2^ mean pairwise distance (i.e. nucleotide diversity).

**Table 4:** Population genetic and molecular evolution statistics for individual RRV genomic segments and coding regions.

Title	Product	Diversity Value ^1^	Total Sites	Segregated Sites	PPS ^2^	Tajima D
RRV_RNA1	NT ^a^	0.0219 ± 0.0005	7092	1467	0.2069	−1.5231
RRV_RNA2	NT	0.0170 ± 0.0004	2314	477	0.2061	−2.0112 *
RRV_RNA3	NT	0.0135 ± 0.0002	1595	539	0.3379	−2.5840 *
RRV_RNA4	NT	0.0151 ± 0.0003	1570	364	0.2318	−2.2123 *
RRV_RNA5	NT	0.0292 ± 0.0005	1695	368	0.2171	−1.0489
RRV_RNA6	NT	0.0161 ± 0.0003	1503	344	0.2289	−2.1731 *
RRV_RNA7	NT	0.0189 ± 0.0003	1707	343	0.2009	−1.8502
RRV_RNA1_CDS	CDS ^b^	0.0220 ± 0.0005	6894	1377	0.1997	−1.4210
RRV_RNA2_CDS	CDS	0.0178 ± 0.0004	1941	376	0.1937	−1.7497
RRV_RNA3_CDS	CDS	0.0135 ± 0.0002	954	149	0.1562	−1.7038
RRV_RNA4_CDS	CDS	0.0152 ± 0.0003	1086	240	0.2210	−2.1088 *
RRV_RNA5_CDS	CDS	0.0303 ± 0.0005	1404	296	0.2108	−0.7728
RRV_RNA6a_CDS	CDS	0.0222 ± 0.0006	190	48	0.2526	−1.7119
RRV_RNA6b_CDS	CDS	0.0104 ± 0.0002	702	97	0.1382	−1.9404
RRV_RNA7_CDS	CDS	0.0183 ± 0.0003	1398	234	0.1674	−1.4037
RRV_RNA1_AA	AA ^c^	0.0041 ± 0.0001	2297	164	0.0714	-
RRV_RNA2_AA	AA	0.0074 ± 0.0001	646	160	0.2477	-
RRV_RNA3_AA	AA	0.0076 ± 0.0001	317	46	0.1451	-
RRV_RNA4_AA	AA	0.0065 ± 0.0001	361	99	0.2742	-
RRV_RNA5_AA	AA	0.0225 ± 0.0002	467	76	0.1627	-
RRV_RNA6b_AA	AA	0.0113 ± 0.0001	233	40	0.1717	-
RRV_RNA7_AA	AA	0.0157 ± 0.0001	465	62	0.1333	-

^1^ This value represents the mean pairwise distance among all pairs for the nucleotide and coding sequences (i.e. nucleotide diversity) and for the amino acid sequences. ^a^ NT = nucleotide sequence, ^b^ CDS = nucleotide coding sequence, ^c^ AA = amino acid sequence. ^2^ proportion of polymorphic sites (PPS = segregated sites/total sites). * ≤0.05.

## Data Availability

The RRV genome sequences generated in this study have been deposited in GenBank under the accession numbers listed in Supplementary Table S1. Raw high-throughput sequencing data are available under [SRA BioProject/PRJNA1399988].

## Supplementary Materials

The following supporting information can be downloaded at: https://www.mdpi.com/article/10.3390/v18101069/s1, Table S1: All RRV isolates and their respective metadata that was utilized throughout this study. Table S2: BEAST phylogenetic analysis output data extracted from the program Tracer for each segment of RRV.
